# Operative Management of Acute Mesenteric Venous Thrombosis With Extensive Intestinal Ischemia: A Case Report

**DOI:** 10.7759/cureus.90892

**Published:** 2025-08-24

**Authors:** Albion Totsi, Eirini Chalkidou, Sotirios Dinas, Elissavet Symeonidou, Christos Papavasileiou

**Affiliations:** 1 Surgery, Papageorgiou General Hospital, Thessaloniki, GRC; 2 General Surgery, Princess Royal University Hospital, London, GBR

**Keywords:** damage control surgery, factor v leiden, intestinal ischemia, mesenteric venous thrombosis, operative treatment

## Abstract

Acute mesenteric venous thrombosis (MVT) is a life-threatening emergency, as delayed treatment may result in bowel ischemia and high mortality. Diagnosis is often challenging due to non-specific symptoms, and management may be conservative or surgical. We report the case of a 52-year-old man who presented to the emergency department with abdominal pain and diarrhea. Although abdominal computed tomography (CT) did not confirm MVT, persistent clinical suspicion and the presence of metabolic acidosis prompted an exploratory laparotomy. Intraoperatively, extensive intestinal ischemia was identified, and given the patient’s hemodynamic instability, damage control surgery was performed, including resection of necrotic bowel, temporary stoma formation, and temporary abdominal closure using the “sandwich” technique. After stabilization in the ICU, a second-look operation allowed for jejunocolic anastomosis, stoma reversal, and definitive abdominal closure. A postoperative venous-phase CT scan confirmed MVT. The patient was discharged after 13 days on anticoagulation, with subsequent diagnosis of Factor V Leiden thrombophilia. This case underscores the importance of maintaining high clinical suspicion for MVT despite inconclusive imaging, as timely surgical intervention can be life-saving.

## Introduction

Acute mesenteric ischemia is a life-threatening emergency with a reported mortality rate of 50%-70%, largely due to delays in diagnosis caused by its non-specific symptoms and diverse clinical presentations [1]. It can be classified into three types: non-occlusive mesenteric ischemia, arterial occlusive mesenteric ischemia, and mesenteric venous thrombosis (MVT) [2]. MVT accounts for approximately 5%-10% of cases and involves thrombosis of the superior mesenteric venous system [3].

The most common presenting symptom of acute MVT is abdominal pain, though its location, severity, and duration may vary depending on the extent of bowel involvement. Other possible features include vomiting, abdominal distension, and diarrhea [4]. Diagnosis is often challenging due to these non-specific findings. Contrast-enhanced abdominal computed tomography (CT) is valuable for assessing bowel viability, while CT angiography with delayed venous phase is considered essential for confirming MVT [4,5]. Treatment depends on the presence of bowel ischemia. Patients without signs of necrosis can be managed conservatively with bowel rest, anticoagulation, and close observation. However, if bowel necrosis or peritonitis is suspected, urgent surgical intervention is indicated [3,6,7].

We present a case of acute MVT with extensive intestinal ischemia and atypical clinical features, highlighting the importance of early suspicion and timely surgical management for improving outcomes.

## Case presentation

**Figure 1:**
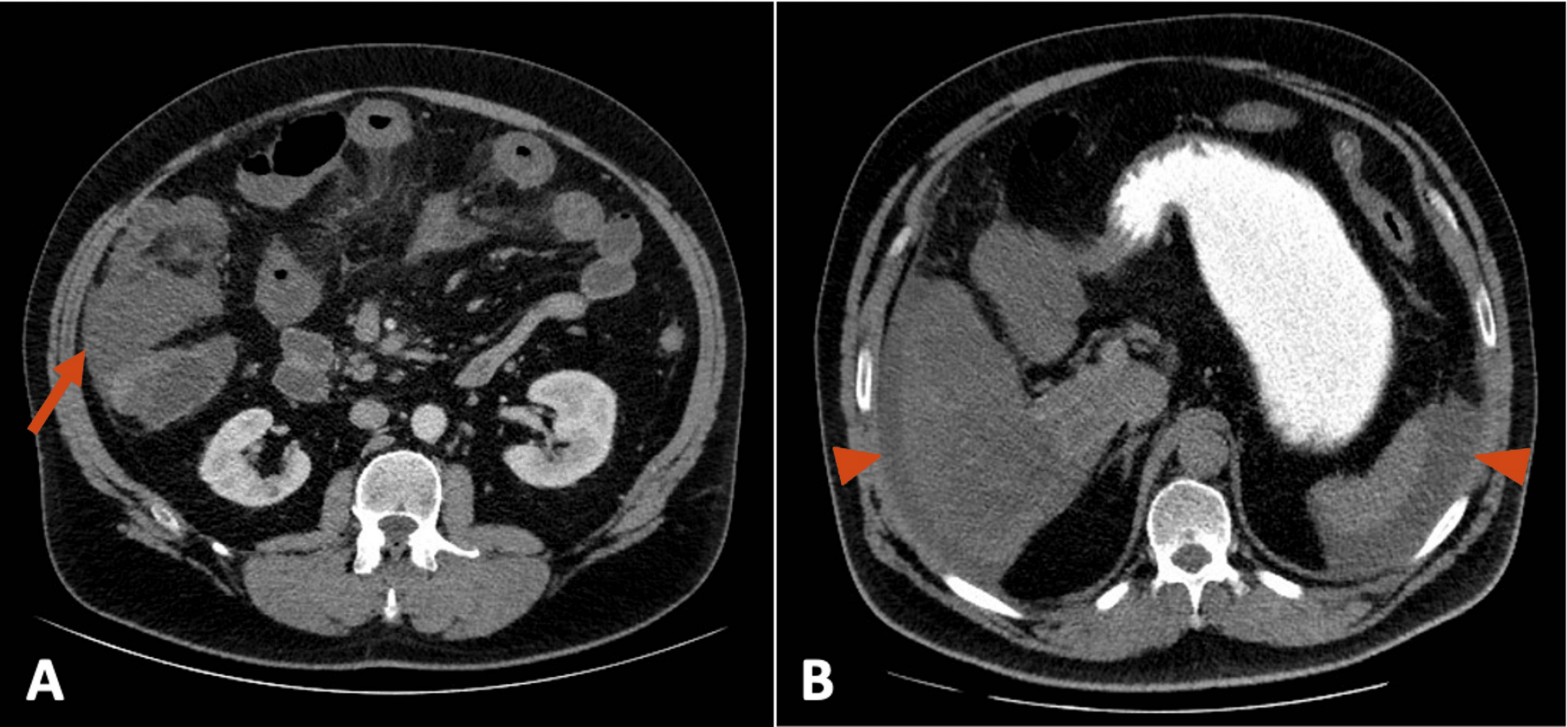
Preoperative abdominal CT scan. (A) Contrast-enhanced image showing edema and thickening of the cecal wall and ileocolic valve (arrow), with associated ileal loop distension. (B) Oral-contrast image demonstrating free peritoneal fluid (arrowhead).

A 52-year-old man presented to the emergency department with diffuse abdominal pain and diarrhea for the past two days. He had no significant past medical history and was not on any regular medications. On examination, the abdomen was distended and diffusely tender, with absent bowel sounds. Digital rectal examination was unremarkable. Laboratory studies showed leukocytosis of 15.10 K/μL (reference range: 3.70-9.50) with 84.2% neutrophils, and elevated C-reactive protein (CRP) at 9 mg/dL (reference range: <0.5). Abdominal CT with oral and IV contrast (arterial phase only) demonstrated marked edema and thickening of the cecal wall and ileocolic valve, mimicking a tumor-like lesion, along with ileal distension and free peritoneal fluid (Figure 1).

In the absence of a definitive diagnosis, the patient was admitted to the surgical department for close observation and further investigation. A few hours later, despite stable vital signs, arterial blood gas analysis revealed mild acidosis and elevated lactate levels. Combined with the high clinical suspicion of peritonitis, this prompted the decision to proceed with exploratory laparotomy.

**Figure 2:**
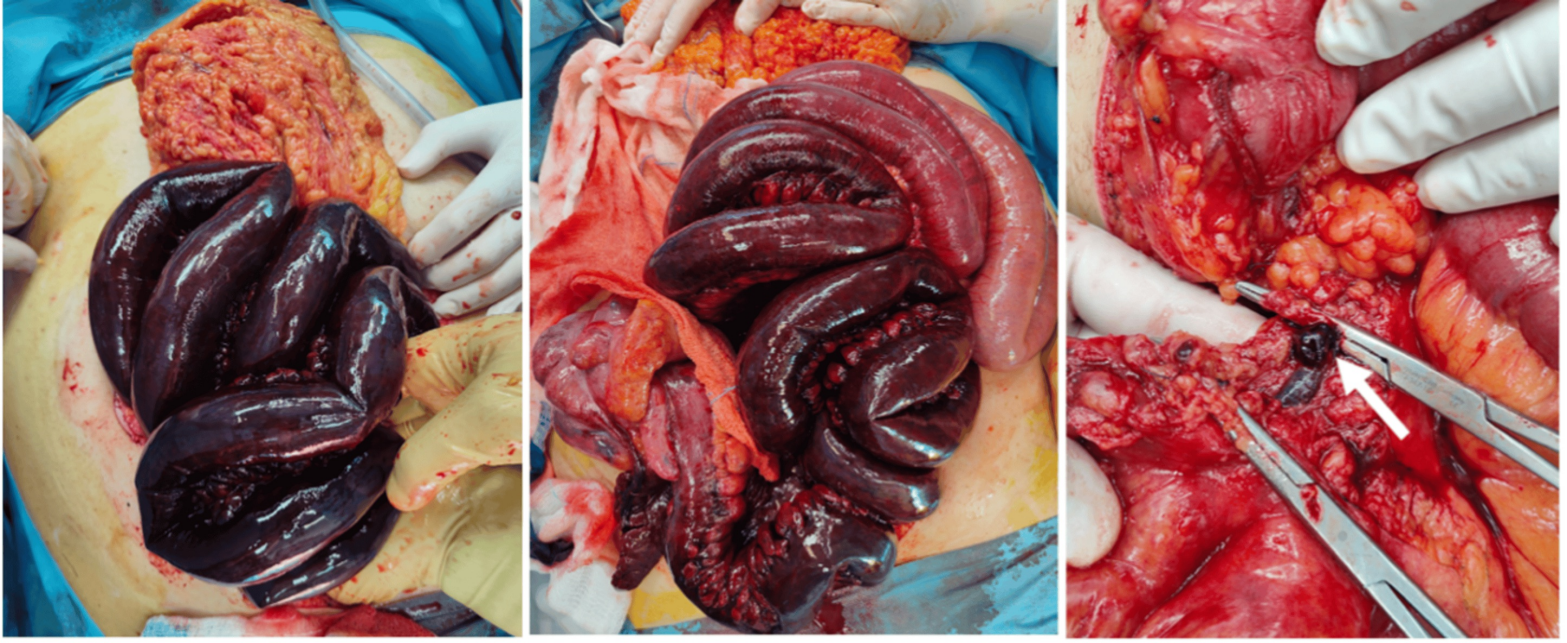
Intraoperative findings of extensive ischemia involving the ascending colon, cecum, ileum, and distal jejunum, showing thrombi within the central mesenteric veins (arrow).

Intraoperatively, extensive ischemia was found involving the ascending colon, cecum, ileum, and distal jejunum. Thrombi were noted within the central mesenteric veins, consistent with superior mesenteric vein thrombosis (Figure 2). All necrotic bowel was resected, leaving approximately 160 cm of the small intestine. Given the uncertain viability of the remaining bowel, a temporary jejunostomy was created instead of performing an anastomosis.

**Figure 3:**
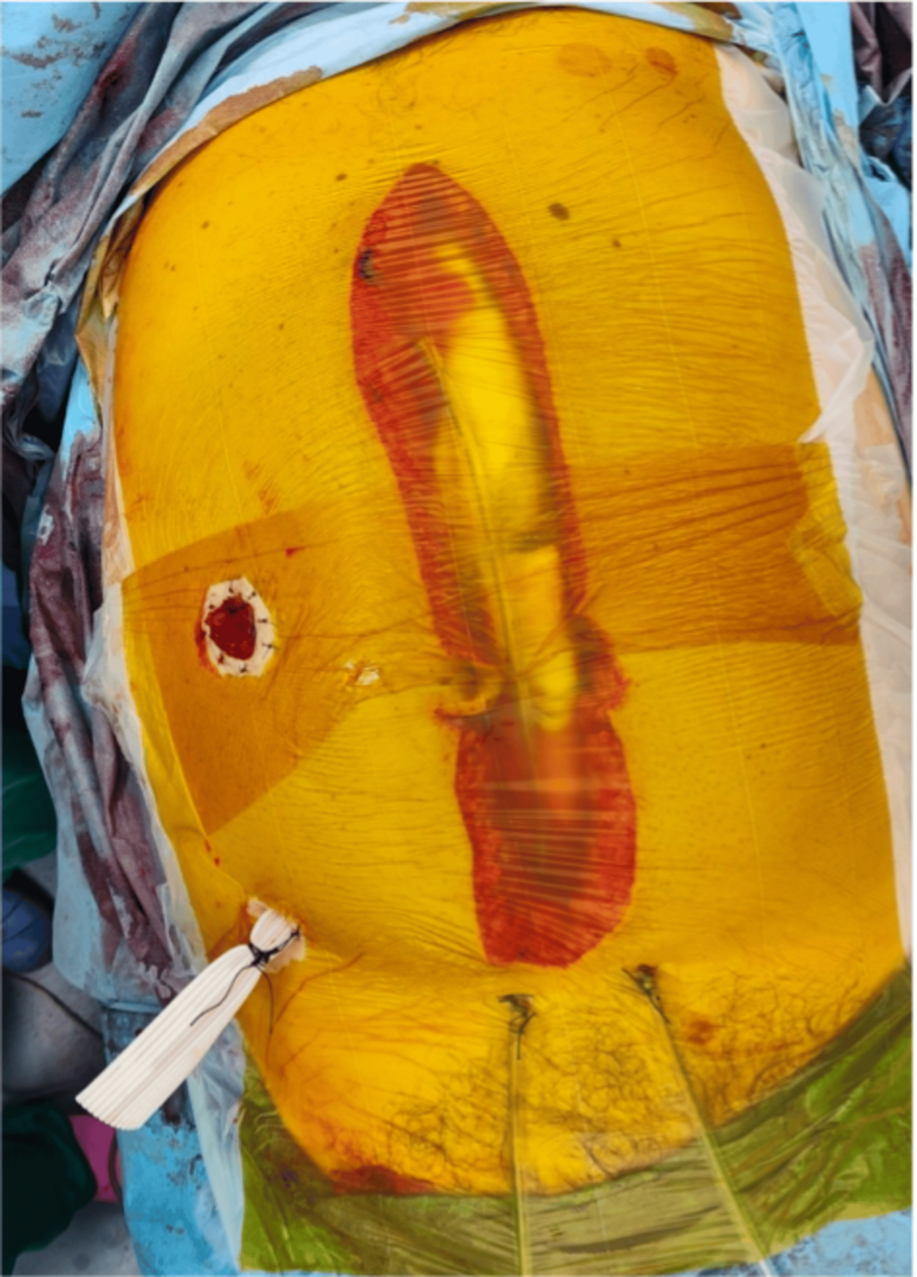
Temporary abdominal closure using the “sandwich” technique.

The abdomen was temporarily closed for second-look surgery using the “sandwich technique” (Figure 3). Soft abdominal packs were placed and covered with sterile drapes on the underside to shield the viscera. Two drainage tubes were positioned above the packs and connected to suction, after which additional sterile drapes were applied to seal the open abdomen, creating a negative-pressure “sandwich.” The patient was then transferred to the ICU for resuscitation in an unstable hemodynamic condition, with lactic acidosis and vasopressor support.

The patient was returned to the operating theater 48 hours later, once stabilized. The remaining bowel appeared viable, with no additional pathological findings. A jejunocolic anastomosis was therefore performed following stoma reversal, and the abdomen was definitively closed. After three days in the ICU, he was transferred back to the surgical department.

**Figure 4:**
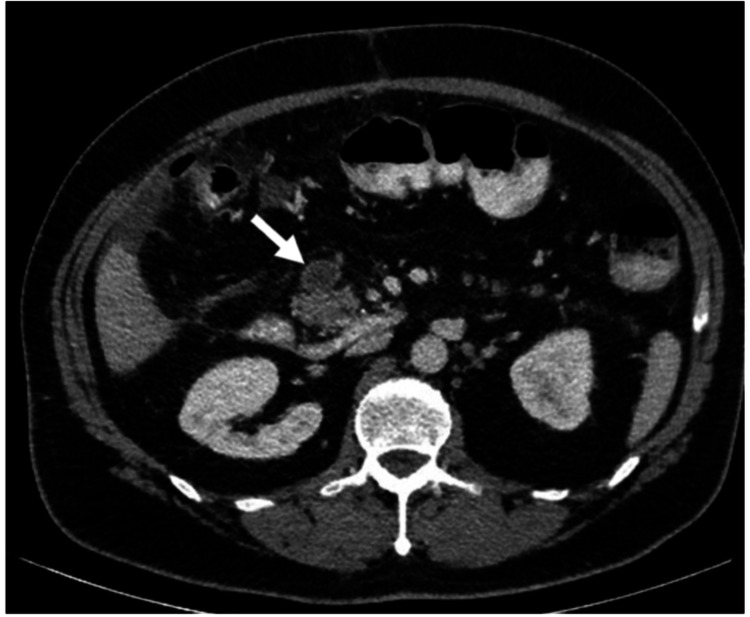
Abdominal CT scan in the portal phase demonstrating complete thrombosis of the superior mesenteric vein (arrow).

A postoperative abdominal CT scan with portal phase was performed to assess the extent of mesenteric thrombosis. It revealed complete thrombosis of the superior mesenteric vein and intrahepatic branches of the portal vein (Figure 4). Anticoagulation therapy with enoxaparin, initiated after the first operation, was continued, and the patient was evaluated by a hematologist. Testing for thrombophilia confirmed heterozygosity for Factor V Leiden. The patient’s condition gradually improved, with the main issue being short bowel syndrome. This was managed with medications to slow intestinal transit (loperamide, paracetamol-codeine) and a specialized diet prescribed by the hospital nutritionist. After 10 days in our unit, he was discharged in good general condition, with dietary guidance and outpatient follow-up. At one month, the patient reported satisfactory bowel function and had returned to daily activities. Anticoagulation was switched from low-molecular-weight heparin to rivaroxaban, which was advised for lifelong use. A follow-up CT scan was also planned to evaluate progression or recanalization of the thrombosis.

## Discussion

The pathophysiologic mechanism of intestinal ischemia begins with acute thrombotic occlusion of the superior mesenteric vein (SMV) and venous arcades, preventing venous return and increasing venous pressure. This results in profound edema of the bowel wall, which gradually leads to submucosal hemorrhage and transmural infarction [8]. In our case, edema and small bowel distension were the only CT findings that could be correlated with bowel infarction, but the impression of a tumor-like lesion of the cecum and the absence of established necrosis gave us confusing diagnostic criteria. Furthermore, the arterial-only phase on the CT scan was not sufficient to show the SMV thrombosis.

On clinical examination, most patients present with generalized abdominal or epigastric tenderness associated with abdominal distension. In cases of delayed diagnosis, signs of peritonitis such as fever and hemodynamic instability may be present [4]. Our case showed diffuse abdominal tenderness, which was not indicative of any specific disease, and the confusing CT findings made the diagnosis more difficult. However, the acidosis and the elevated lactate levels in the arterial blood gases were critical in our decision to perform an exploratory laparotomy, even though there were no obvious signs of bowel necrosis on imaging modality.

In some cases of isolated SMV thrombosis, bowel perfusion may be compensated for by the presence of collateral flow. On the other hand, when the mesenteric-portal axis is involved, there is a high risk of necrosis of large segments of the small bowel [2,3]. Intraoperative findings demonstrated thrombosis not only of the central mesenteric veins but also of the terminal branches, resulting in complete occlusion of the venous drainage. The extent of the thrombosis was evident on postoperative abdominal CT scan with portal phase, occupying the SMV up to the intrahepatic branches of the portal vein.

Damage control surgery is strongly indicated in cases of intestinal ischemia where resection of large bowel segments is required and patients are hemodynamically unstable. Second-look surgery is necessary when the viability of the bowel is uncertain, allowing time for resuscitation and improvement of bowel perfusion. Re-exploration is usually performed after 48 hours, when definitive decisions about anastomosis and permanent abdominal closure can be made [7,9]. In our case, we followed a damage control strategy with a rapid resection of the right colon and a large part of the small bowel, which had a necrotic appearance. No anastomosis was performed; instead, a colostomy was created, and the abdomen was left open for second-look surgery. The sandwich technique was chosen as a simple, easy-to-apply, and quick option for temporary abdominal closure. The advantages of the method are low cost, availability, and the ability to minimize heat and fluid loss in a measurable and controlled manner [10].

Coagulopathy is the main pathogenic factor in MVT, although in 20% of cases no specific cause is identified. Conditions such as malignancies, inflammatory diseases, and antiphospholipid syndrome can lead to hypercoagulability, while inherited disorders like Factor V Leiden mutation or deficiencies of protein C or protein S may result in thrombophilia [11,12]. Our patient had no history of malignancy or inflammatory disease, so he needed to be screened for other hypercoagulable conditions. Tests for thrombophilic defects revealed heterozygosity for Factor V Leiden, which is a mutation of one of the clotting factors. Given the increased risk of recurrent thrombosis, the patient was advised to remain under close hematology follow-up for long-term management of anticoagulant therapy [13].

## Conclusions

The diagnosis of MVT and intestinal ischemia is very challenging due to non-specific symptoms and clinical signs. In high clinical suspicion, a CT scan with portal phase will confirm the diagnosis. If there is any indication of intestinal necrosis, an exploratory laparotomy is necessary, while in critically ill patients who require reassessment of bowel viability, a damage control strategy should be followed. Patients without a history of coagulopathy should be thoroughly investigated for thrombophilic defects to prevent recurrent thrombosis.
